# Accurate Prediction of Absorption Spectral Shifts of Proteorhodopsin Using a Fragment-Based Quantum Mechanical Method

**DOI:** 10.3390/molecules26154486

**Published:** 2021-07-25

**Authors:** Chenfei Shen, Xinsheng Jin, William J. Glover, Xiao He

**Affiliations:** 1 Shanghai Engineering Research Center of Molecular Therapeutics and New Drug Development, School of Chemistry and Molecular Engineering, East China Normal University, Shanghai 200062, China; chenfeishen@foxmail.com (C.S.); xsjinleo@163.com (X.J.); 2 NYU Shanghai, 1555 Century Avenue, Shanghai 200122, China; william.glover@nyu.edu; 3 NYU-ECNU Center for Computational Chemistry at NYU Shanghai, Shanghai 200062, China; 4 Department of Chemistry, New York University, New York, NY 10003, USA

**Keywords:** proteorhodopsin, absorption spectrum, fragmentation QM method, electric field

## Abstract

Many experiments have been carried out to display different colors of Proteorhodopsin (PR) and its mutants, but the mechanism of color tuning of PR was not fully elucidated. In this study, we applied the Electrostatically Embedded Generalized Molecular Fractionation with Conjugate Caps (EE-GMFCC) method to the prediction of excitation energies of PRs. Excitation energies of 10 variants of Blue Proteorhodopsin (BPR-PR105Q) in residue 105GLN were calculated with the EE-GMFCC method at the TD-B3LYP/6-31G* level. The calculated results show good correlation with the experimental values of absorption wavelengths, although the experimental wavelength range among these systems is less than 50 nm. The ensemble-averaged electric fields along the polyene chain of retinal correlated well with EE-GMFCC calculated excitation energies for these 10 PRs, suggesting that electrostatic interactions from nearby residues are responsible for the color tuning. We also utilized the GMFCC method to decompose the excitation energy contribution per residue surrounding the chromophore. Our results show that residues ASP97 and ASP227 have the largest contribution to the absorption spectral shift of PR among the nearby residues of retinal. This work demonstrates that the EE-GMFCC method can be applied to accurately predict the absorption spectral shifts for biomacromolecules.

## 1. Introduction

The rhodopsin proteorhodopsin and related proteins have aroused continuous and extensive interest both experimentally and theoretically among researchers, especially during the past 20 years [1,2,3,4,5]. Rhodopsin is a seven transmembrane α-helices (TM) protein which uses retinal as a chromophore. Retinal, the aldehyde of vitamin A and a polyene chromophore, is derived from β-carotene and utilized in the all-trans/13-cis configurations in microbial rhodopsins allowing certain microorganisms to convert light into metabolic energy. 11-cis/all-trans retinal acts as the molecular basis of animal vision and is attached by a Schiff base linkage to the conserved residue lysine (LYS231) sidechain in the middle of TM7. The retinal Schiff base (RSB) is protonated (RSBH+) in most cases [6].

Proteorhodopsin(PR), as a member of the microbial rhodopsin family, is a light-driven protein found in marine proteobacteria. Due to the widespread global distribution of proteobacteria in sea water, PR may have an important effect on global solar energy input in the biosphere [7,8]. The maximum absorption wavelength of PR is tuned according to the depth at which the bacteria live [9,10]. It is well known that a shorter wavelength of the light corresponds to a higher energy and larger power to penetrate deeper water. To date, many experiments have been carried out to display different colors of PR and its mutants, but the mechanism of color tuning of PR is still not fully elucidated.

Borhan and co-workers performed spectral tuning of all-trans-retinal PRs, in which one or multiple residues were mutated by rational mutagenesis, enabling the absorption maximum of the pigment in the range of 425 to 644 nm [11]. In addition, for the study of single point mutation, previous works demonstrated that one of the determinants for color tuning of PR is at position 105, GLN in Blue-absorbing PR (BPR—PR105Q) and LEU in Green-absorbing PR (GPR—PR105L) [10]. Kandori and co-workers successfully introduced several amino acid mutations at position 105 of BPR (PR105Q) and investigated the absorption properties. High-performance liquid chromatography analysis showed that the isomeric composition of the all-trans form was greater than 70% for all mutants [12].

The effect of mutations on the optical properties of PRs is usually difficult to predict, and thus it would be desirable to employ theoretical calculations to aid in the rational design of PRs with tailored photo-physical properties [13]. Unfortunately, modeling the excitation energy and other excited-state properties of PRs is incredibly challenging due to the necessity of accurate quantum mechanical (QM) excited-state calculation (which scales steeply with the system size) and the large system size involved.

Recently, our group developed the Electrostatically Embedded Generalized Molecular Fractionation with Conjugate Caps (EE-GMFCC) method for quantitatively characterizing properties of proteins with localized excitations (i.e., involving a single chromophore). The excitation energy, transition dipole moment, and oscillator strength of wild-type Green Fluorescent Protein (GFP) calculated by EE-GMFCC were found to be in excellent agreement with results of full system time-dependent density functional theory (TDDFT) [13]. In this study, we applied the EE-GMFCC method for the accurate prediction of excitation energies of PRs. We hope that this method can be used to help investigate the mechanism of color tuning in PRs, and for the rational design of its mutagenesis.

## 2. Computational Approaches

The ground state of microbial rhodopsin possesses an all-trans configuration for its chromophore [6]. Our previous study demonstrated that the protein environment plays an important role in the excited-state properties of GFP [13]. We therefore expect a similar dependence on protein environment for the electronic properties of the PR chromophore. To handle the full complexities of the PR chromophore-protein interactions, we use Electrostatic Embedded (EE)-GMFCC, which is an extension of GMFCC to include many-body environment effects in each fragment QM calculation using embedding charges that represent the remaining fragments [14,15].

### 2.1. The EE-GMFCC Method for Excited State and Its Application for PR105Q

Before applying the EE-GMFCC method to absorption spectrum calculations of PRs, we give a brief description of this quantum fragmentation approach to large biomolecular systems. The EE-GMFCC method was initially developed for the total energy calculation on the ground state of large proteins [14,15,16]. In the EE-GMFCC framework, a protein with *N* residues is divided into *N-2* fragments with each residue capped by its neighboring residues (conjugate caps) [14,15,17]. Each fragment’s energy is computed at the QM level. Interactions between fragments that are not directly bonded are handled by including two-body interactions in larger fragments formed by two residues in close contact within a distance cutoff. Typically, three-body and higher-order interactions within the EE-GMFCC scheme are small and can be neglected. The QM energies of the conjugated caps are deducted to avoid overcounting contributions from overlapping regions of the fragments.

The ground-state energy of a protein is calculated by EE-GMFCC as follows,

(1) $$\begin{array}{c} E_{\mathrm{EE}-\mathrm{GMFCC}}^{\mathrm{Ground}\;\mathrm{State}}=\overset{N-1}{\underset{i=2}{\sum }}\tilde{E}({\mathrm{Cap}}_{i-1}^{*}A_{i}{\mathrm{Cap}}_{i+1})-\overset{N-2}{\underset{i=2}{\sum }}\tilde{E}({\mathrm{Cap}}_{i}^{*}{\mathrm{Cap}}_{i+1})+\overset{N-3}{\underset{i=1}{\sum }}\overset{N}{\underset{\begin{array}{c} j=i+3\\ \left|R_{ij}\right|\leq \lambda_{2} \end{array}}{\sum }}\left({\tilde{E}}_{ij}-{\tilde{E}}_{i}-{\tilde{E}}_{j}\right)\\ -\left\{\overset{N-2}{\underset{i=2}{\sum }}\underset{m\in k_{i}}{\sum }\underset{n\in l_{i}}{\sum }\frac{q_{m\left(k_{i}\right)}q_{n\left(l_{i}\right)}}{R_{m\left(k_{i}\right)n\left(l_{i}\right)}}-\overset{N-3}{\underset{i=1}{\sum }}\underset{m^{\prime }\in i}{\sum }\overset{N}{\underset{\begin{array}{c} j=i+3\\ \left|R_{ij}\right|\leq \lambda_{2} \end{array}}{\sum }}\underset{n^{\prime }\in j}{\sum }\frac{q_{m^{\prime }\left(i\right)}q_{n^{\prime }\left(j\right)}}{R_{m^{\prime }\left(i\right)n^{\prime }\left(j\right)}}\right\} \end{array}$$

where $\tilde{E}$ denotes the sum of the self-energy of a fragment and the interaction energy between the fragment and its background charges. ${\mathrm{Cap}}_{i-1}^{*}A_{i}{\mathrm{Cap}}_{i+1}$ represents the *i*th residue (A_i_) covalently bonded with molecular caps of ${\mathrm{Cap}}_{i-1}^{*}$ and ${\mathrm{Cap}}_{i+1}$. The definition of molecular caps is given in ref. [14]. The first two sums calculate the one-body (1B) QM energy of the system, which accounts for the covalently-linked three neighboring residues. The third term in Equation (1) represents the two-body (2B) QM energy corrections, arising from the two non-covalently-linked residues that are spatially in close contact. The last two terms are applied to subtract the double-counted interaction between QM region and background charges in all 1B and 2B calculations. $\left|R_{ij}\right|$ is the minimum distance between any two atoms from residues *i* and *j*. $\lambda_{2}$ is the cutoff distance of 2B. $q_{m\left(k_{i}\right)}$ represents the charge of the *m*th atom of fragment *k_i_*.$\;R_{m\left(k_{i}\right)n\left(l_{i}\right)}$ represents the distance between atoms $m\left(k_{i}\right)$ and $n\left(l_{i}\right)$. For a detailed description of the total ground-state energy calculation of proteins using the EE-GMFCC method, please refer to our previous work [14].

Recently, our group extended the ground state EE-GMFCC method to make it applicable for predicting the properties of excited states of the luminescent biomolecule GFP [13]. The calculation of excitation energy, transition dipole moment, and oscillator strength using EE-GMFCC showed good agreement with the corresponding full system QM results. In this study, based on our previous works [13,18,19,20,21], the three-body QM interaction term was neglected because it only slightly improves the accuracy but with substantially more computational time. Moreover, the cutoff distance threshold for the two-body QM interactions was set to 4 Å to strike a compromise between attained accuracy and computational cost.

In its current formulation, the EE-GMFCC method is appropriate only for local excitations, wherein the dominant electronic response following excitation is localized on a single molecular unit, i.e. a chromophore. Such a local excitation can be defined by the excited state of the chromophore in isolation being qualitatively similar to its excited state in the protein environment. We expect this to be the case for PRs, because its non-standard residue LYR231 has been identified as the chromophore responsible for light absorption.

After we define the locally excited region, fragments including this region are defined as excited fragments (EF), whereas fragments excluding the excited region are defined as unexcited fragments (UEF). Then the total excited-state energy of the protein can be described as the summation of the excited state energy $E_{\mathrm{EE}-\mathrm{GMFCC}}^{\mathrm{Excited}-\mathrm{State}}\left(\mathrm{EF}\right)$ from the contributions of excited fragments and the ground-state energy $E_{\mathrm{EE}-\mathrm{GMFCC}}^{\mathrm{Excited}-\mathrm{State}}\left(\mathrm{UEF}\right)$ from unexcited fragments as follows:

(2) $$\begin{array}{ll} E_{\mathrm{EE}-\mathrm{GMFCC}}^{\mathrm{Excited}\;\mathrm{State}}\left(EF\right) & =\overset{m+1}{\underset{i=m-1}{\sum }}{\tilde{E}}^{\prime }({\mathrm{Cap}}_{i-1}^{*}A_{i}{\mathrm{Cap}}_{i+1})\\ & -\overset{m}{\underset{i=m-1}{\sum }}{\tilde{E}}^{\prime }({\mathrm{Cap}}_{i}^{*}{\mathrm{Cap}}_{i+1})+\overset{N}{\underset{\begin{array}{c} j=1\\ \left|R_{mj}\right|\leq \lambda_{2}\\ j\notin \left[m-2,m+2\right] \end{array}}{\sum }}\left({\tilde{E}}^{\prime }{}_{mj}-{\tilde{E}}^{\prime }{}_{m}-{\tilde{E}}_{j}\right)\\ & -\left\{\overset{N-2}{\underset{i=2}{\sum }}\underset{m_{0}\in k_{i}}{\sum }\underset{n\in l_{i}}{\sum }\frac{q_{m_{0}\left(k_{i}\right)}^{\prime }q_{n\left(l_{i}\right)}^{\prime }}{R_{m_{0}\left(k_{i}\right)n\left(l_{i}\right)}}-\overset{N-3}{\underset{i=1}{\sum }}\underset{m^{\prime }\in i}{\sum }\overset{N}{\underset{\begin{array}{c} j=i+3\\ \left|R_{ij}\right|\leq \lambda_{2} \end{array}}{\sum }}\underset{n^{\prime }\in j}{\sum }\frac{q_{m^{\prime }\left(k_{i}\right)}^{\prime }q_{n^{\prime }\left(l_{i}\right)}^{\prime }}{R_{m^{\prime }\left(k_{i}\right)n^{\prime }\left(l_{i}\right)}}\right\} \end{array}$$

Most denotations in Equation (2) (the EE-GMFCC method for excited state) are similar to the ground state calculation. Here we just point out the key differences. The superscript “prime” represents the excited-state energy of the subsystem containing the localized excitation region (residue *m*). In addition, for the unexcited fragments, the energy calculation is the same as the ground state. Then the total EE-GMFCC energy at the excited state is given by:

(3) $$E_{\mathrm{EE}-\mathrm{GMFCC}}^{\mathrm{Excited}\;\mathrm{State}}=E_{\mathrm{EE}-\mathrm{GMFCC}}^{\mathrm{Excited}\;\mathrm{State}}\left(\mathrm{EF}\right)+E_{\mathrm{EE}-\mathrm{GMFCC}}^{\mathrm{Excited}\;\mathrm{State}}\left(\mathrm{UEF}\right)$$

Here, we assume that the atomic charges of the protein at the excited state are approximated to be the same as those at the ground state. We subtract Equation (1) from Equation (3) to obtain the excitation energy of the protein based on the EE-GMFCC approach. The final expression of the EE-GMFCC excitation energy is as follows:

(4) $$\omega =\overset{m+1}{\underset{i=m-1}{\sum }}\omega ({\mathrm{Cap}}_{i-1}^{*}A_{i}{\mathrm{Cap}}_{i+1})-\overset{m}{\underset{i=m-1}{\sum }}\omega ({\mathrm{Cap}}_{i}^{*}{\mathrm{Cap}}_{i+1})+\overset{N}{\underset{\begin{array}{c} j=1\\ \left|R_{mj}\right|\leq \lambda_{2}\\ j\notin \left[m-2,m+2\right] \end{array}}{\sum }}\left(\omega_{mj}-\omega_{m}\right)$$

In this study, *m* is the residue number of chromophore LYR. ω stands for the excitation energy. The QM region is given in the brackets including two kinds of fragments, namely ${\mathrm{Cap}}_{i-1}^{*}A_{i}{\mathrm{Cap}}_{i+1}$ and ${\mathrm{Cap}}_{i}^{*}{\mathrm{Cap}}_{i+1}$, whereas the remaining part of the protein was represented by background atomic charges. The QM calculations were performed using the Terachem package [22,23,24]. ${\mathrm{Cap}}_{i-1}^{*}A_{i}{\mathrm{Cap}}_{i+1}$ represents the QM region of the *i*th residue (*A_i_*) covalently bonded with molecular caps of ${\mathrm{Cap}}_{i-1}^{*}$ and ${\mathrm{Cap}}_{i+1}$. The definition of molecular caps is given in ref. [14]. The first two summations of Equation (4) calculate the one-body (1B) QM contributions to the total excitation energy of the system.

**Figure 1:**
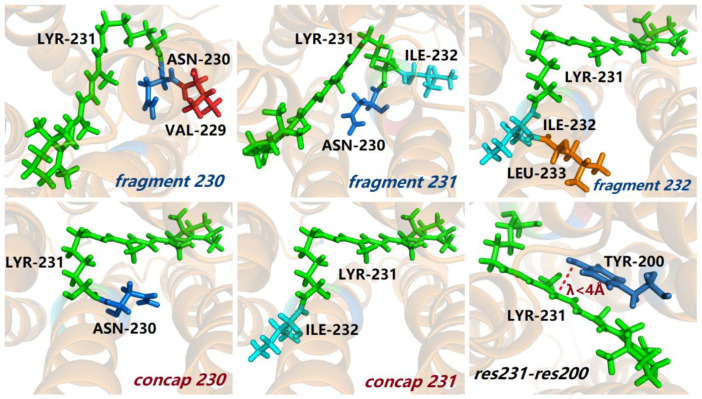
Graphical representation of fragments, concaps, and 2B QM interaction. The background α-helix cartoon of the PR protein with 80% transparency is described by background charges, whereas the sticks of the corresponding residues represent the QM region in the EE-GMFCC calculation. The green sticks denote the chromophore LYR231. The structures all come from the same configuration of the PR protein.

There are two kinds of fragment interactions in Equation (4), which includes one-body (1B) and two-body (2B) QM interaction terms. The one-body (1B) term represents the fragment with sequentially connected two residues or three residues, whereas the two-body (2B) QM interaction term represents the interaction between two non-neighboring residues that are spatially in close contact within a distance threshold. In this study, for PRs, the 1B term includes the chromophore LYR231 and its neighboring residues, namely, Fragment(230), Fragment(231), Fragment(232), Concap(230), and Concap(231), where Fragment(230), Fragment(231), and Fragment(232) contain residues 229–231, 230–232, and 231–233, respectively, and Concap(230) and Concap(231) contain residues 230–231 and 231–232, respectively (see Figure 1 and Figure S1 of the Supplementary Materials). The 2B term in this study represents the QM interaction between LYR231 and non-neighboring residue that is within 4 Å of LYR231. Therefore, Equation (4) becomes:

(5) $$\begin{array}{c} \omega =\omega_{1B}+\omega_{2B}\\ =\omega \left(\mathrm{Fragment}\left(230\right)\right)+\omega \left(\mathrm{Fragment}\left(231\right)\right)+\omega \left(\mathrm{Fragment}\left(232\right)\right)-\omega \left(\mathrm{Concap}\left(230\right)\right)\\ -\omega \left(\mathrm{Concap}\left(231\right)\right)+\overset{N}{\underset{\begin{array}{c} j=1\\ \left|R_{231,j}\right|\leq 4Å\\ j\notin \left[229,233\right] \end{array}}{\sum }}\left(\omega_{231,j}-\omega_{231}\right) \end{array}$$

where $\omega_{1B}$ and $\omega_{2B}$ denote the 1B and 2B QM interactions, respectively. The last term $\overset{N}{\underset{\begin{array}{c} j=1\\ \left|R_{231,j}\right|\leq 4Å\\ j\notin \left[229,233\right] \end{array}}{\sum }}\left(\omega_{231,j}-\omega_{231}\right)$ is the sum of 2B QM corrections, namely, the excitation energy between LYR231 and its non-neighboring residue *j*, if the distance between any pair of atoms from LYR231 and residue *j* is less than or equal to 4 Å. In the 1B term, the interaction between the chromophore and non-neighboring residues is described by the embedding electrostatic charges. The interactions between the chromophore and non-neighboring residues in spatially close contact are subjected to quantum mechanical treatment in the 2B QM calculations.

### 2.2. Structure Preparation and Molecular Dynamics (MD) Simulation

#### 2.2.1. Homology Modelling

**Figure 2:**
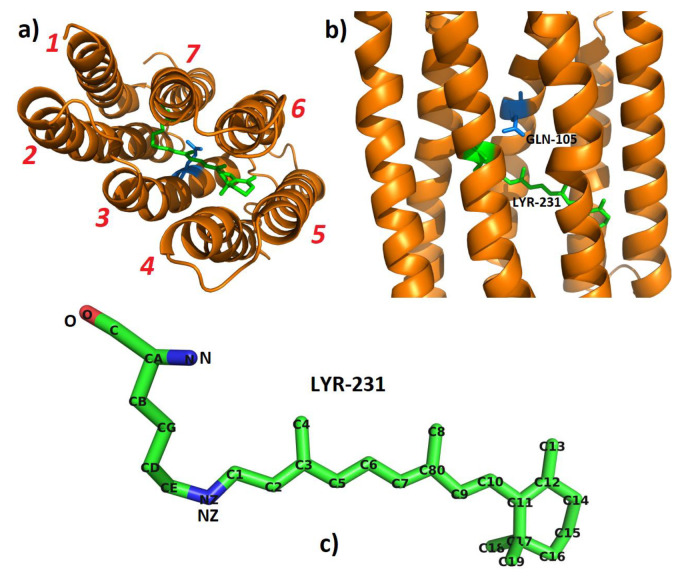
(**a**) The top view of PR. (**b**) The side view of PR. In (**a**,**b**), the green moiety denotes the chromophore, and the blue moiety is residue 105. (**c**) The structure of LYR and all hydrogens were removed for simplicity. All atom names are marked for clarity.

In this work, the structure of Blue-absorbing PR (BPR—PR105Q) was prepared by means of a hybrid Quantum Mechanics/Molecular Mechanics (QM/MM) approach and MD simulation [25]. The initial structure was taken from the X-ray crystal structure of BPR (PR105Q) in the dark state (PDB id: 4JQ6, chain B), which we chose as the template of PR105Q (PR with GLN105). The MODELLER [26] software, was utilized for homology modelling of PR105Q. The alignment of PR105Q and chain B of 4JQ6 was performed using TM-align [27]. Residue LYS231 and retinal were combined in a protonated retinal Schiff base (RSBH+) linkage as a non-standard residue in the Amber package [28,29], which we named LYR231 in this work. The structure of BPR (PR105Q) and LYR231 are shown in Figure 2. The distances between the chromophore and all residues are given in Supplementary Figure S2 of the Supplementary Materials.

#### 2.2.2. Force Field Construction of the Non-Standard Residue LYR231

To make direct comparison to experiment, absorption spectra of the wild-type protein and several other mutated proteins were computed under high pH conditions, such that the ASP97 residue near the Schiff base was deprotonated. For the structure of chromophore, a LYS residue jointed with retinal (named LYR) was formed by the Schiff base at the connection site. The isomeric all-trans configuration occupied more than 70% for almost all of the mutant proteins. Therefore, we chose the all-trans isomer as the initial structure of LYR (see Figure 2) [12].

We set the residue ASP97 at the deprotonated state. The experimental value of the absorption peak compared in this study was the measured value when the protein was solvated in high pH solution. The Amber18 program was utilized to obtain the force-field parameters [29]. The parameters of chromophore were created using the Generalized Amber Force Field (GAFF) [30]. The Amber ff14SB force field and the TIP3P water model [31] were used for other parts of the protein [32] and water, respectively. Force-field parameters of non-standard residue (LYR231, the chromophore) were obtained from the ANTECHAMBER module with the AM1-BCC charge model [33] using the semi-empirical quantum mechanics (sqm) method [34].

#### 2.2.3. Molecular Dynamics (MD) Simulation

The initial protein structure was solvated in a cubic periodic box of TIP3P water molecules with each side at least 10 Å from the nearest solute atom, and the total size of the cubic box was 87.298 × 71.649 × 57.894 Å^3^. The counter ions Na^+^ were added to neutralize the entire system according to its total net charge. The molecular structure of PR105Q was optimized under molecular mechanics using two steps. First, the protein was restrained and all other molecules were relaxed. Next, the entire system was energy minimized. Each minimization procedure consisted of 10,000 steps of the steepest descent optimization, followed by 40,000 steps of the conjugate gradient optimization approach.

We used a set of configurations extracted from simulations to compute the averaged absorption spectra using QM calculations. The approach based on the sequential use of simulations and quantum mechanical calculations (denominated Sequential QM/MM) has been widely used to calculate spectroscopic properties of molecules in liquid environments [35]. A wide variety of works use this sequential method to predict absorption spectra and other properties, such as NMR and emission spectra, supplying converged results similar to the experimental results.

To obtain the ensemble-average absorption spectra, conformations of PR105Q were extracted from Quantum Mechanics/Molecular Mechanics (QM/MM) molecular dynamics (MD) simulation trajectories [28,36,37]. The QM/MM MD simulation of PR was carried out using Amber18 [38] interfaced to the Gaussian09 package [39]. The QM part, consisting of the residue LYR and residues 105 and 200, was treated with the M06-2X functional in conjunction with the 6-31G* basis set [40].

Schapiro and co-workers studied the initial excited state dynamics of GPR, and their simulations indicated that the retinal-TYR200 interaction played an important role in the outcome of the photo isomerization [25]. In this study, during classical MD simulation, the fluctuation of TYR200 had a significant influence on the excitation energy of the chromophore LYR231. Therefore, we performed MD simulation of PR using the QM/MM method, and TYR200 was included in the QM region.

The detailed procedure of MD simulation is given as follows. First, the optimized system was heated to 300 K in 50 ps. Secondly, a 500 ps equilibration run with classical MD was carried out before the final 10 ps QM/MM MD simulation for a production run at 300 K [41]. Langevin dynamics with a collision frequency of 1.0 ps^−1^ was used to control the temperature. A 25 Å cutoff was applied to the QM/MM electrostatic interactions, and the SHAKE algorithm was applied to restrain bonds with hydrogen atoms.

The QM/MM MD simulations were performed for PR105Q and nine other mutants [28,29]. A total of 100 snapshots were evenly extracted from the trajectory of the last ps QM/MM MD simulation with a time interval of 10 fs. Each conformation of the selected 100 snapshots was subsequently calculated by the EE-GMFCC method to obtain the vertical excitation energy (Equation (5)) [13]. Here we chose the B3LYP density functional in TDDFT calculations [42].

#### 2.2.4. Key Residue Mutation and Fragment-Based QM Calculations for Excitation Energies

Residue 105 plays an important role in determining the color of PR, where BPR (PR105Q) and GPR (PR105L) have GLN and LEU at this position, respectively [12]. Accurate predictions of absorption spectral shifts upon point mutations are critical to the rational mutagenesis design of PR [11,43,44,45]. Here, residue 105 is a vital residue that affects the absorption spectral shift of PR. Kandori and co-workers carried out an experimental investigation in which several different point mutations were introduced for the color determining residue 105 [12].

For the QM calculations, water molecules were removed from the conformation of MD simulation trajectory. The excitation energy of the protein was calculated with the EE-GMFCC method at the TD-B3LYP/6-31G* level [46,47] using Equation (5).

**Figure 3:**
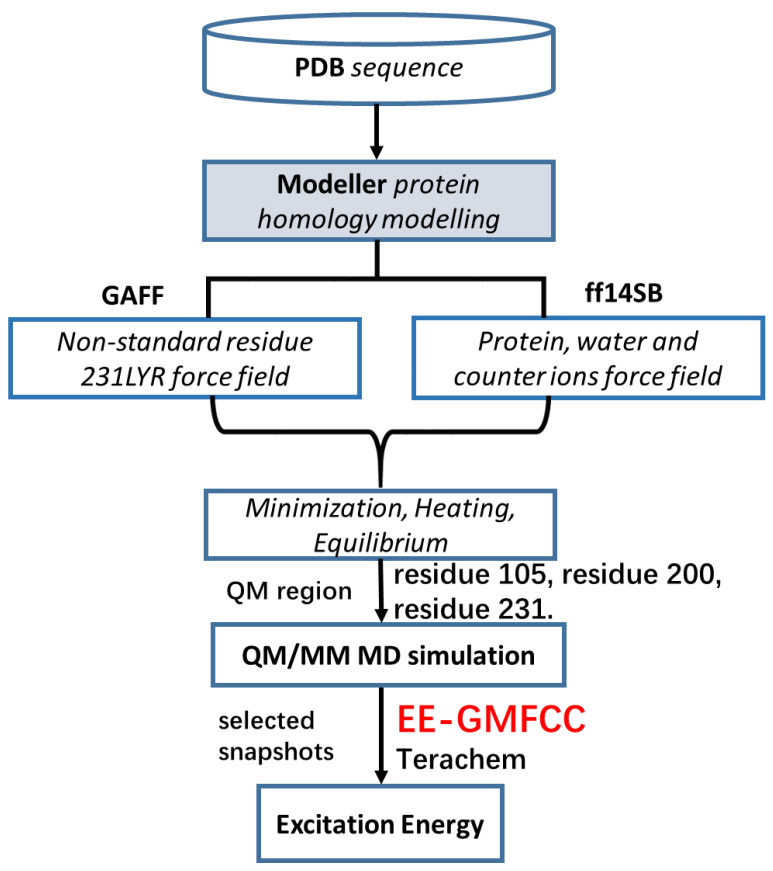
The work flow of theoretical calculations for PR105Q and its nine mutations in this study. Residue 105 was mutated to nine other residues.

Figure 3 shows the work flow of the complete computational protocol utilized in this study for model construction, MD simulation, and excitation energy calculations. We used Ambertools to complete mutation operation for PR. First, we removed sidechain atoms of residue 105, while the backbone atoms (C, N, CA, O) of residue 105 and other parts of the protein were reserved. Second, we changed the residue name of R105 to the name of the residue to be mutated. Third, the sidechain atoms of the new residue were added to the backbone of R105 by the Amber program. Finally, the added residue was energy minimized to avoid unreasonable repulsive interactions with nearby residues. The minimization process was undertaken using Amber18 [28].

## 3. Results and Discussion

### 3.1. Comparison between Calculated Excitation Energies and Experiment for Wild-Type PR and Its Mutants

From experimental investigation, residue 105 is an important amino acid that influences the absorption spectrum of the chromophore in PR. In this study, we mutated residue 105 to other amino acids for predicting the absorption spectral shift. Vertical excitation energies were calculated on mutated PRs using the EE-GMFCC method. Predicted excitation energies were compared with corresponding experimental absorption peaks.

**Figure 4:**
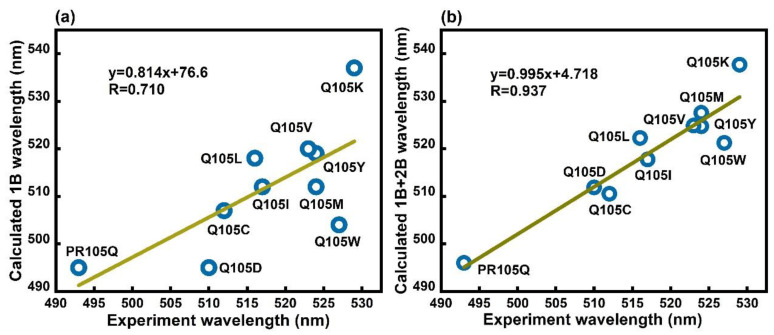
The correlation between the experimental absorption wavelengths of different mutations of PR105Q and calculated results using EE-GMFCC. The 1B and 1B + 2B results are both provided. (**a**) 1B: only the one-body QM interactions are included. (**b**) 1B + 2B: both the one-body and two-body QM interactions are included (see Equation (4)). 1B and 2B represent the excitation energy contributions from the covalently-linked three neighboring residues, and the two non-covalently-linked residues that are spatially in close contact, respectively.

Figure 4 shows that the correlation coefficient (*R*) between the EE-GMFCC results (1B + 2B) and experimental values is 0.937, and the equation of linear regression is *y* = 0.995*x* + 4.718. In contrast, the results given by EE-GMFCC without two-body (2B) QM interaction corrections yielded a worse agreement with the experiment. The correlation coefficient given by EE-GMFCC with 1B correction is merely 0.710 (see Figure 4a), indicating that, perhaps unsurprisingly, the residues which are spatially in close contact with the central chromophore have the greatest impact on the excitation energy of the chromophore in PR. From Table 1, we can see that the mean unsigned error (MUE) of the predicted excitation energies of those 10 systems decreases from 8.0 to 3.5 nm calculated by EE-GMFCC from 1B to 1B + 2B, compared to experimental values. This is direct evidence that the absorption wavelength of the chromophore is affected by its surrounding chemical environment.

**Figure 5:**
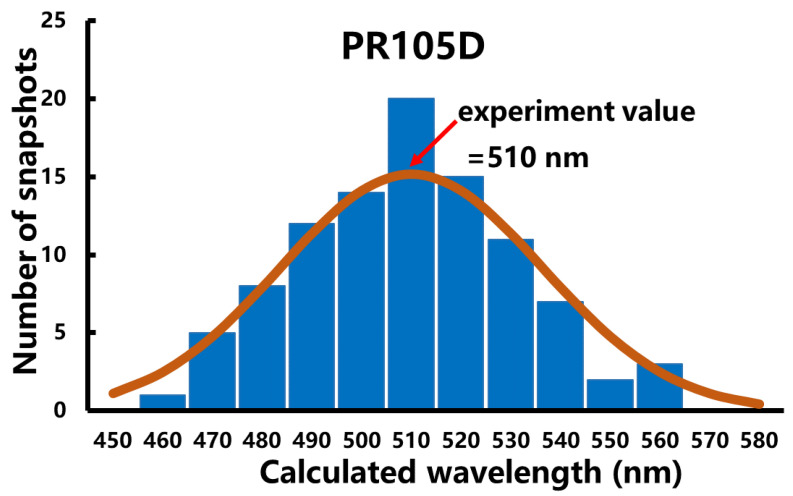
Distribution of calculated absorption wavelengths for one hundred conformations of PR105D extracted from the last ps of QM/MM MD trajectory. The average value of calculated results is 512 nm, which is in excellent agreement with the experimental value of 510 nm.

One hundred snapshots extracted from the last ps of QM/MM MD trajectory were selected to calculate the average excitation energy of PR. The excitation energy distribution of these 100 conformations of PR105D is shown in Figure 5. As shown in Figure 5, the conformations with the predicted excitation energy close to the experimental absorption peak have the largest population. The most probable absorption wavelength is almost equal to the average value, which is in good agreement with the experimental data [12]. Excitation energy distributions for the other nine mutants are given in Supplementary Figure S4 of the Supplementary Materials.

### 3.2. Residue-Based Decomposition of Excitation Energies

Next, we studied the excitation energy contribution from each residue around the chromophore. To avoid interference from embedding charges, we used the GMFCC scheme, which turns off the background charges in each fragment QM calculation, because 1B and 2B QM interactions in EE-GMFCC include many-body environmental effects through electrostatic embedding.

The GMFCC scheme leads directly to the excitation energy contribution from each residue around the chromophore LYR231 by subtracting the single chromophore excitation energy from the two-body (residue-chromophore) excitation energy [48]. Our previous work used the GMFCC method to provide a qualitative prediction of the relative shift that each residue contributes to the excitation energy of the GFP chromophore [13]. Here, we applied the same approach, in which the 2B distance threshold was set to 4 Å between the chromophore and nearby residues. The excitation energy contribution of each residue around the chromophore was calculated by GMFCC based on 100 snapshots from QM/MM MD simulation. The excitation energies were also calculated at the TD-B3LYP/6-31G* level for consistency.

**Figure 6:**
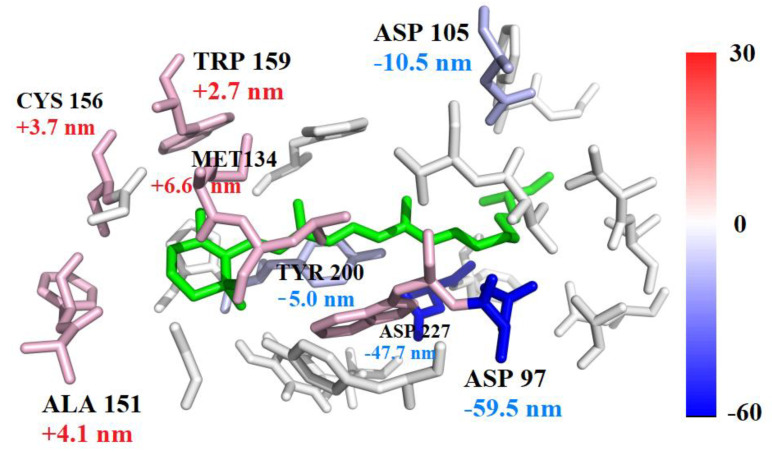
Excitation energy contributions of 2B QM interactions involving residues close to chromophore are presented by the colors blue and red, where the color gradient in units of nm is scaled based on the 2B QM contribution, and the distance threshold λ for 2B correction was set to 4 Å. Residues with positive 2B QM correction have a blue shift of the absorption spectrum and are colored blue, whereas residues with negative 2B QM correction have a red shift and are colored red.

The per-residue decomposition of the average excitation energy of the PR105D protein is shown in Table 2 and Figure 6. Residues spatially close to chromophore had the greatest influence on the excitation energy of the protein. ASP97, ASP105, and ASP227 yield the largest blue shifts, whereas MET134, PHE152, and LEU135 show red shifts of PR105D. As shown in Figure 6, the most blue-shifted residue is ASP97, yielding a wavelength shift of −59.5 nm (+267.9 meV), compared to the chromophore alone, whereas the most red-shifted residue is MET134, with a wavelength shift of +6.6 nm (−26.3 meV). 

### 3.3. The Local Electric Field along the Retinal

It is known that the polyene chain of the retinal chromophore has considerable charge-transfer character in its lowest excited state, which we confirm below. It is therefore reasonable to expect that point mutations of the protein can modulate the excitation energy of the retinal through changes in the electrostatic field. To test this idea, we investigated the electric field along the polyene chain under different point mutations. A measure of the electric field along the polyene chain is given by [49,50,51]:

(6) $$E_{c10-c1}=\frac{\sum_{i=1,i\notin LYR}^{N}\frac{1}{4\pi \varepsilon }\left(\frac{q_{i}}{\left|{\vec{r}}_{i}-{\vec{r}}_{c10}\right|}-\frac{q_{i}}{\left|{\vec{r}}_{i}-{\vec{r}}_{c1}\right|}\right)}{\left|{\vec{r}}_{c10}-{\vec{r}}_{c1}\right|}$$

where $E_{c10-c1}$ is the electric field induced by protein residues from C1 to C10 on the conjugate chain, which approximates the electric field along the retinal chain (see Figure 2). The term i∉LYR denotes that charges of residue LYR231 were excluded from the calculation of electric field. $q_{i}$ represents the atomic charge of the *i*th atom, ${\vec{r}}_{i}$ denotes the coordinate vector of *i*th atom, and $\left|{\vec{r}}_{i}-{\vec{r}}_{c10}\right|$ represents for the distance between atom *i* and atom C10.

**Figure 7:**
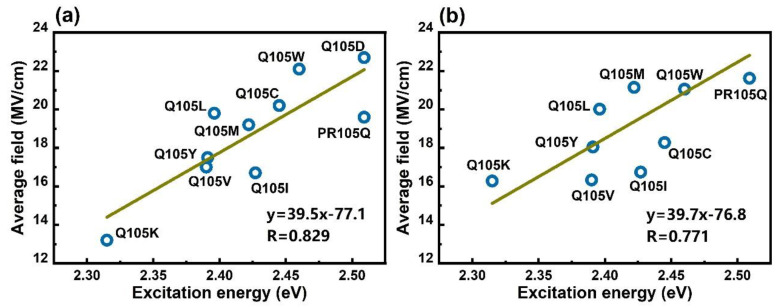
Correlation between the average electric field of 100 snapshots taken from QM/MM MD simulation trajectory for PR105Q and nine mutants, and the calculated 1B QM excitation energy by EE-GMFCC. (**a**) The average electric field calculated by the Amber ff14SB charge model. (**b**) The average electric field calculated by the PPC charge model.

The 1B excitation energies calculated by the EE-GMFCC method show good correlation with the average electric field based on the Amber ff14SB force field [32] and polarized protein-specific charge (PPC) [49,52,53,54] models for the 100 snapshots extracted from the QM/MM MD simulation (see Table 3 and Figure 7). The correlation coefficient (*R*) between 1B excitation energies and the electric fields from the Amber ff14SB charge model is 0.829, and the equation of linear regression is *y* = 39.5*x* − 77.1. In comparison, the correlation coefficient (*R*) between 1B excitation energies and the electric fields from the PPC charge model is 0.771, and the equation of linear regression is *y* = 39.7*x* − 76.8. The correlations between the average electric fields (with the Amber and PPC charge models) and 2B excitation energies calculated by EE-GMFCC are shown in Supplementary Figure S7 of the Supplementary Materials, and the correlations between the average electric fields (with the Amber and PPC charge models) and experimental excitation energies are shown in Supplementary Figure S8 of the Supplementary Materials.

**Figure 8:**
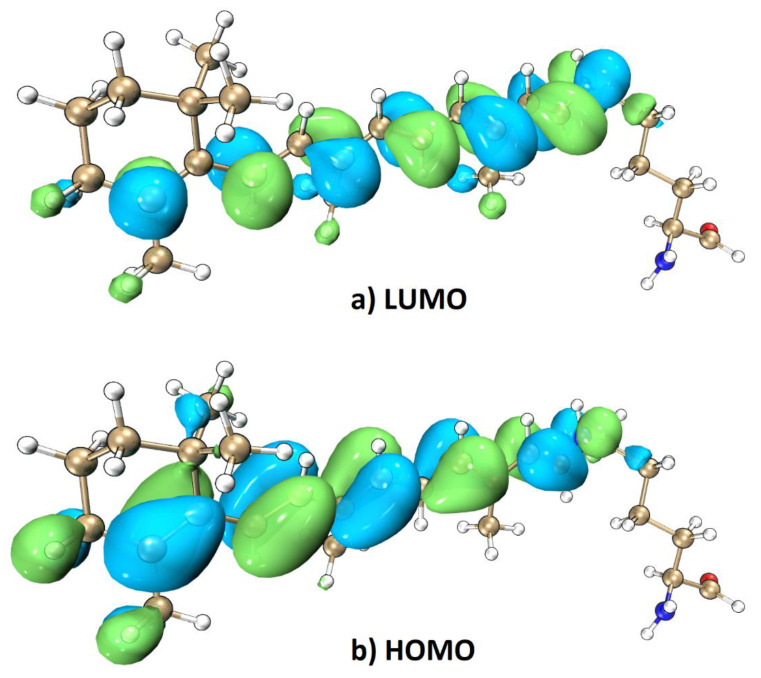
Representative snapshot of PR105D from QM/MM MD simulation and calculated LUMO (**a**) and HOMO (**b**) orbitals at the TD-B3LYP/6-31G* level where LYR231 was subjected to quantum mechanical treatment with background charges of the remaining part of PR105D. Charge transfer process of electron excitation from HOMO to LUMO [55,56].

**Figure 9:**
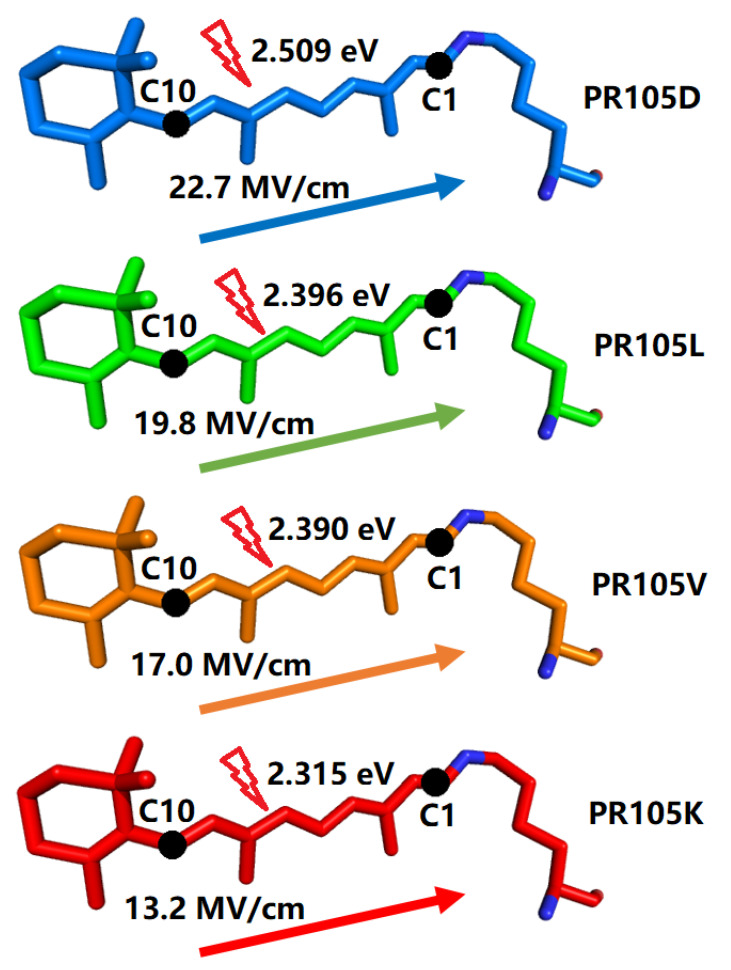
The calculated average electric fields and excitation energies of EE-GMFCC-1B for 100 snapshots extracted from QM/MM MD simulations of four representative mutations, namely, PR105D, PR105L, PR105V, and PR105K.

Figure 8 shows that the HOMO magnitude decreases from C10 to C1, whereas the LUMO magnitude decreases in the reverse direction to that of the HOMO. From natural transition orbital (NTO) analysis, the HOMO and LUMO are the dominant orbitals involved in this excitation. The electron excitation from the HOMO to LUMO of the chromophore thus has considerable charge-transfer character. Based on the direction of charge transfer along the polyene chain, we expect that increasing the environmental electric field along the conjugate chain from C10 to C1 will result in a blue shift of the electronic excitation [43]. Conversely, decreasing the magnitude of the electric field will cause a red shift of the electronic excitation. This expectation is borne out in our results: Figure 7 shows that the largest electric field between C10 and C1, with a value of 22.7 MV/cm (with the Amber force field), is observed for PR105D with a corresponding 1B excitation energy of 2.509 eV, which is larger than PR105L with 19.8 MV/cm. Similarly, the smallest electric field of 13.2 MV/cm is observed for PR105K, which has the smallest 1B excitation energy of 2.315 eV. A higher strength of the electric field corresponds to a higher excitation energy, which yields a blue shift (see Figure 9).

As shown in Figure 9, the electric fields of PR105D (22.7 MV/cm), PR105L (19.8 MV/cm), PR105V (17.0 MV/cm), and PR105K (13.2 MV/cm) decrease gradually as the excitation energy progressively decreases in the order of 2.509, 2.396, 2.390, and 2.315 eV. As discussed above, there is a certain linear correlation between the electric field and excitation energy. It is worth noting that a prediction of the excitation energy change upon point mutation based on an electric field calculation is much faster compared to the ab initio EE-GMFCC calculations, although the correlation is not perfect in this study. The possible cause of such deviation might arise from the inaccuracy of the charge model used in the electric field calculations. To investigate the effect of the charge model on the electric field calculations of PRs, we tested the PPC charge model for comparison with the Amber ff14SB charge model [32]. The PPC model takes the polarization-induced effect of the protein into consideration by assigning a polarized atomic charge to each atom using the self-consistent RESP [57,58] fitting scheme for each amino acid, with the rest of the protein acting as an electrostatic field. Here, we utilized the PPC method to refit the atomic charges of the protein, and applied the PPC charge model to the PR system for each electric field calculation. Further details of the PPC charge fitting scheme are provided in Ref. [59]. The fitted PPC charges replace the original Amber charges for each atom of the PR systems, and the electric field is calculated using Equation (6). The results of EE-GMFCC-2B and a comparison of performance between Amber and PPC charge models are given in Supplementary Figures S7 and S8 of the Supplementary Materials. In general, the performance of the PPC model is not superior to the result predicted by the Amber ff14SB charge model, indicating that a more sophisticated method might need to be applied to make electric field-excitation energy correlations for quantitative accuracy for PR systems.

## 4. Conclusions

In this study, we applied the Electrostatically Embedded Generalized Molecular Fractionation with Conjugate Caps (EE-GMFCC) method to predict the excitation energy of PRs. Excitation energies of wild-type PR and its nine mutants were calculated with the EE-GMFCC method at the TD-B3LYP/6-31G* level over hundreds of thermally sampled snapshots from ab initio QM/MM molecular dynamics simulations.

The calculated excitation energies show good correlation with the experimental values of absorption wavelengths despite the fact that the experimental wavelengths among these ten systems vary by less than 50 nm. The correlation coefficient (*R*) between the EE-GMFCC results (1B + 2B) and experimental values is 0.937. In contrast, the results calculated by EE-GMFCC without two-body QM interaction corrections yield poorer agreement with the experiment. The correlation coefficient given by EE-GMFCC with 1B corrections was merely 0.710, indicating that the residues which are spatially in close contact with the central chromophore have the greatest impact on the excitation energy of the chromophore in PR.

We also utilized the GMFCC method to decompose the excitation energy contributions of residues near the chromophore. The most blue-shifting residue is ASP97, which yields a −59.5 nm (+267.9 meV) wavelength shift in average, whereas the most red-shifting residue is MET134 with a +6.6 nm (−26.3 meV) wavelength shift. The overall spectral shift of the 2B QM correction on PR mutants was small, mainly due to a cancellation between blue-shifting and red-shifting residues.

The calculated excitation energies using the EE-GMFCC method with 1B corrections show good correlations with the predicted average electric field using the Amber and PPC charge models, and the correlation coefficients (*R*) between them are 0.829 and 0.771, respectively. Predicting the excitation energy change based on the average electric field could be an alternative and efficient approach for the rational design of PRs with tailored photo-physical properties. Overall, our results demonstrate that the EE-GMFCC method is a useful tool for accurately and efficiently predicting the excited-state properties of large biological systems.

In this work, a relatively short 1 ps of trajectory data was analyzed because of the two following problems. First, MD simulations using classical force fields do not fully sample the correct configurations of the retinal structure due to the low accuracy of the force field, and we found that the predicted absorption wavelengths of incorrect retinal conformations deviate substantially from the experimental values; thus, a QM/MM approach was critical. Second, QM/MM MD simulations with more than 100 atoms in the QM region are very time consuming, limiting us to trajectories of 10 ps in length, which left 1 ps of production data following equilibration. Although it would have been ideal to use more uncorrelated points for the computation of the excitation energy, given the close comparison with the experiment and the low fluctuations observed, we think that this sampling is sufficient to showcase our methodology.

It is also worth noting that the 1-ps QM/MM MD trajectory that we use for analysis will not sample long timescale protein fluctuations [60]; however, the good agreement found between theoretical excitation energies and experimental measurements suggests that >1-ps fluctuations have a minor influence on the average absorption energy. This finding is in contrast to solvated chromophores, which can have large couplings between the excitation energy and >1-ps solvation dynamics [61,62,63,64]. The origin of this disparity could be due to a relatively conserved environment of the chromophore in the protein matrix, unlike in solvent, although we cannot fully rule out a fortuitous agreement of our results with experiment. The role of long-time fluctuations of the protein on the excitation energies of the PR chromophore is an interesting open question that we hope to address in the future.

## Figures and Tables

**Table 1:** Calculated excitation energy using EE-GMFCC at the TD-B3LYP/6-31G* level for different mutations of PR. “Dev.” stands for the deviation from the experimental value. 1B and 2B denote the excitation energy calculated by 1B correction, and 1B + 2B corrections, respectively. 1B and 2B represent the excitation energy contributions from the covalently-linked three neighboring residues, and the two non-covalently-linked residues that are spatially in close contact, respectively. MUE denotes the mean unsigned error.

Mutation	Exp (nm) *^a^*	1B (nm)	2B (nm)	Dev. (1B)	Dev. (2B)
Q105D	510	495	512	−15	2
Q105W	527	504	521	−23	−6
Q105C	512	507	511	−5	−1
Q105L	516	518	522	2	6
PR105Q	493	495	496	2	3
Q105M	524	512	528	−12	4
Q105Y	524	519	525	−5	1
Q105V	523	520	525	−3	2
Q105I	517	512	518	−5	1
Q105K	529	537	538	8	9
Average				−5.6	2.1
MUE				8.0	3.5

*^a^* Absorption wavelength of experimental value in high pH solution where ASP97 is deprotonated. [12].

**Table 2:** Excitation energy decomposition per residue based on an ensemble average over 100 conformations. The residues are spatially in close contact with the chromophore and their QM contributions are calculated by GMFCC at the TD-B3LYP/6-31G* level. “Ex” represents the excitation energy in eV. ΔEx represents the excitation energy difference between 2B (residue + LYR231) and LYR231. ΔWL represents the wavelength difference between 2B (residue + LYR231) and LYR231 in nm.

Res. Name	Ex (eV)	ΔEx (meV)	ΔWL (nm)
LYR231 *^a^*	2.2348	0.0	0.0
LEU40	2.2329	−1.9	0.5
VAL68	2.2392	4.4	−1.1
THR69	2.2322	−2.6	0.7
ALA72	2.2317	−3.1	0.8
TYR95	2.2326	−2.2	0.5
ASP97	2.5027	267.9	−59.5
TRP98	2.2182	−16.6	4.2
THR101	2.2258	−9.0	2.2
VAL102	2.2265	−8.3	2.1
ASP105	2.2779	43.1	−10.5
MET134	2.2084	−26.3	6.6
LEU135	2.2155	−19.3	4.8
GLY138	2.2395	4.7	−1.2
ALA151	2.2183	−16.4	4.1
PHE152	2.2119	−22.9	5.7
GLY155	2.2345	−0.2	0.1
CYS156	2.2202	−14.6	3.7
TRP159	2.2239	−10.9	2.7
TRP197	2.2344	−0.3	0.1
TYR200	2.2551	20.3	−5.0
PRO201	2.2289	−5.8	1.5
TYR204	2.2308	−4.0	1.0
TYR223	2.2343	−0.5	0.1
ASP227	2.4446	209.8	−47.7
PHE228	2.2364	1.6	−0.4
PHE234	2.2344	−0.4	0.1
GLY235	2.2341	−0.7	0.2

*^a^* With only LYR231 included in the QM region. In other cases, each residue and LYR231 are in the QM region.

**Table 3:** With the Amber and PPC charge models, the calculated average electric field (in MV/cm) between C10 and C1 (see Figure 2) along the polyene chain of chromophore based on the ensemble average over 100 snapshots from QM/MM MD simulation. Experimental values (eV) of excitation energies are given for comparison. 2B represents the two-body corrected QM excitation energy, and 1B represents the one-body QM excitation energy calculated by EE-GMFCC.

Mutations	Exp. (eV)	2B (eV)	1B (eV)	Ave. Field *^a^* (MV/cm)	Ave. Field *^b^* (MV/cm)	Distance (C10-C1)
Q105D	2.436	2.428	2.509	22.7	25.9	9.849
Q105W	2.357	2.382	2.460	22.1	21.1	9.809
Q105C	2.427	2.431	2.445	20.2	18.3	9.799
Q105L	2.408	2.378	2.396	19.8	20.0	9.826
PR105Q	2.520	2.502	2.509	19.6	21.6	9.826
Q105M	2.371	2.353	2.422	19.2	21.1	9.824
Q105Y	2.371	2.365	2.391	17.5	18.0	9.830
Q105V	2.375	2.365	2.390	17.0	16.3	9.850
Q105I	2.403	2.398	2.427	16.7	16.7	9.855
Q105K	2.349	2.311	2.315	13.2	16.3	9.857

*^a^* Average electric field calculated by the Amber ff14SB charge model. *^b^* Average electric field calculated by the PPC charge model.

## Data Availability

The data presented in this study are available in Supplementary Material.

## Supplementary Materials

The following are available online. The fragmentation scheme of the EE-GMFCC method; Analysis of the protein structure; Comparison between the EE-GMFCC and traditional QM/MM calculations; The convergence test of the EE-GMFCC calculations; Excitation energy distributions of 100 conformations of PR105Q and its nine mutants; Correlations between the calculated electric fields (with the Amber and PPC charge models) and excitation energies calculated by EE-GMFCC, and experimental excitation energies.
