# Supplementary material for: Accurate Prediction of Absorption Spectral Shifts of Proteorhodopsin Using a Fragment-Based Quantum Mechanical Method
Source: Molecules. 2021 Jul 25;26(15):4486. doi: 10.3390/molecules26154486 (PMC8347797; doi:10.3390/molecules26154486)
Supplement: Supplementary file 1 [file molecules-26-04486-s001.zip › molecules-1235434-supplementary.pdf]

The Supporting Information for

Accurate Prediction of Absorption Spectral Shifts of Proteorhodopsin Using a Fragment-based Quantum Mechanical Method

*Chenfei Shen[1], Xinsheng Jin[1], William J. Glover[2,3,4] and Xiao He[1,3]**

[1]Shanghai Engineering Research Center of Molecular Therapeutics and New Drug Development, School of Chemistry and Molecular Engineering, East China Normal University, Shanghai, 200062, China

[2]NYU Shanghai, 1555 Century Avenue, Shanghai, 200122, China

[3]NYU-ECNU Center for Computational Chemistry at NYU Shanghai, Shanghai, 200062, China

[4]Department of Chemistry, New York University, New York, New York, 10003, USA

* To whom correspondence should be addressed: xiaohe@phy.ecnu.edu.cn

CONTENT

1. The fragmentation scheme of the EE-GMFCC method.
2. Analysis of the protein structure.
3. Comparison between the EE-GMFCC and traditional QM/MM calculations.
4. The convergence test of the EE-GMFCC calculations.
5. Excitation energy distributions of 100 conformations of PR105Q and its 9 mutants.
6. Correlations between the calculated electric fields (with the AMBER and PPC charge models) and excitation energies calculated by EE-GMFCC, and experimental excitation energies.

1. The fragmentation scheme of the EE-GMFCC method

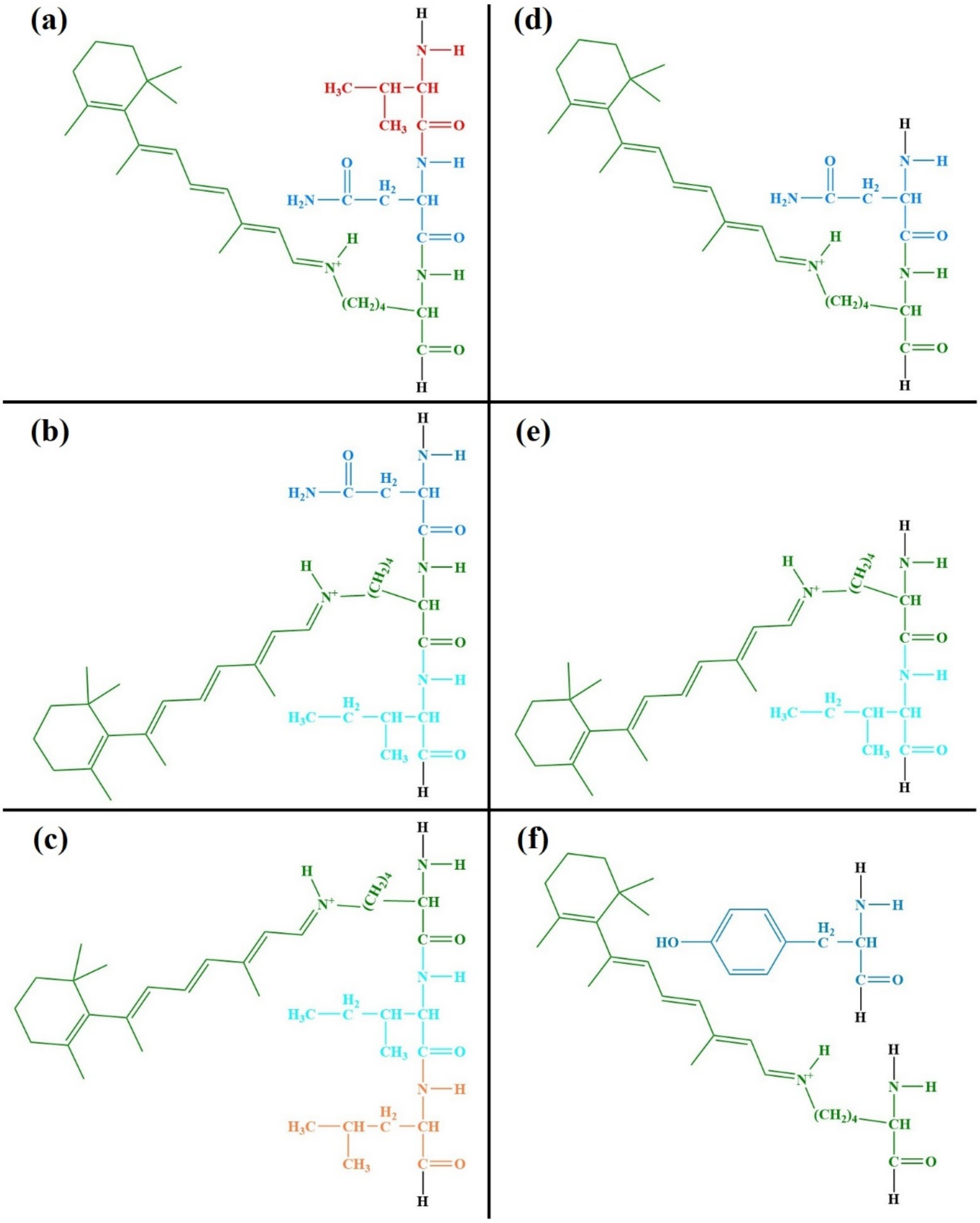

Figure S1. The atomic structures (same as shown in Figure 1) of the EE-GMFCC scheme for fragment, concap and 2B QM interaction. (a) Fragment 230 including VAL-229, ASN-230 and LYR-231 in red, blue and green respectively. The dangling bonds are saturated by hydrogen atoms. (b) Fragment 231. (c) Fragment 232. (d) Concap 230 including ASN-230 and LYR-231. (e) Concap 231. (f) 2B QM interaction between LYR-231 and TYR-200. The remaining atoms of the protein are described by background charges for each fragment QM calculation.

2. Analysis of the protein structure.

The distance between residues R_m and R_n could be measured by the average distance between every pair of atoms of these two residues.

$$\text{Distance}(R_m - R_n) = \frac{\sum_{i=1,\alpha}\sum_{j=1,\beta}\sqrt{\sum_{coor=x,y,z}\{coor[R_{m(i)}] - coor[R_{n(j)}]\}^2}}{\alpha * \beta} \quad (S1)$$

where R_m and R_n are mth and nth residues in the protein, α and β are the atom numbers in R_m and R_n, respectively. $coor[R_{m(i)}]$ represents the cartesian coordinate of ith atom in residue R_m, where *coor* denotes x, y or z.

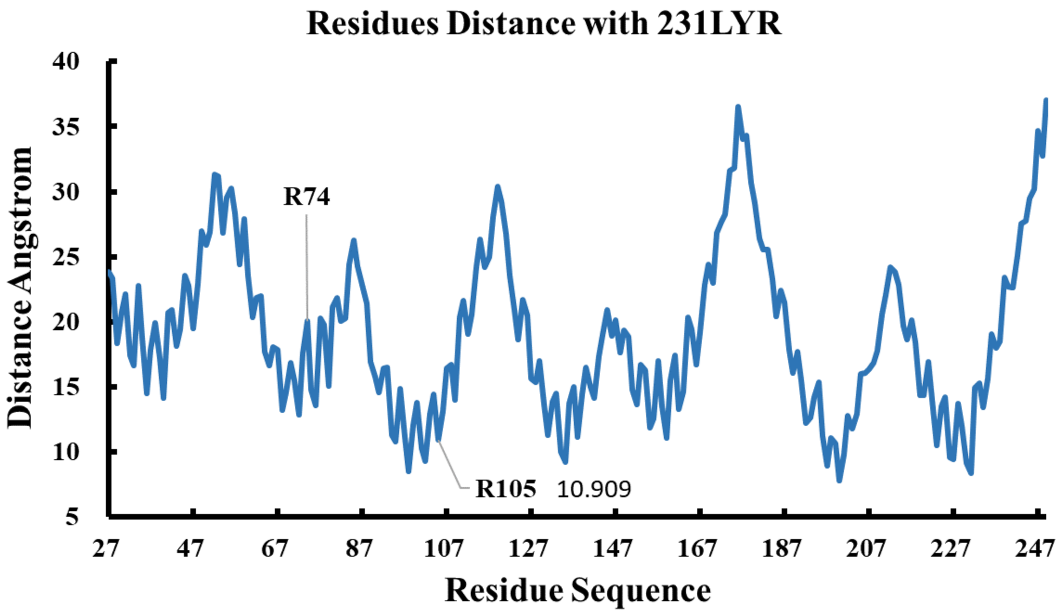

Figure S2. Distance between each residue and LYR231 from residues 27 to 249 of PR105Q.

We can understand the trends in Figure S2 as follows: first, the distances between the surrounding residues and the LYR231 fluctuate periodically for 7 α-helices of PR: there are seven peaks and seven troughs. Second, the troughs correspond to the center of each helix, and the peaks correspond to the end of each helix. Third, the smaller high-frequency fluctuations reflect the orientation of the side chain of each residue, whether it is inside forward or outside forward to the retinal.

3. Comparison between the EE-GMFCC and traditional QM/MM calculations.

We also performed traditional QM/MM calculations on 10 PR105D conformations at the TD-B3LYP/6-31G* level. The QM region in the QM/MM calculations consists of residues 230, 231, 232 and closest non-covalent neighbors within 2.5 Å from the central retinal. As shown in Table S1 and Figure S3, the overall trend of the results of those two calculations is close. However, the EE-GMFCC calculation includes more quantum mechanical interactions between the retinal and the residues within 4.0 Å from it. Therefore, the average deviation between the traditional QM/MM calculations and EE-GMFCC results is 0.06 eV. Meanwhile, as demonstrated in our previous study[1], the EE-GMFCC calculation with the 2B correction shows linear scaling, which results in an expected 10-fold speedup as compared to traditional full-system TDDFT calculations for systems of the size studied in the present work.

Table S1. Calculated excitation energies of the EE-GMFCC approach and traditional QM/MM method for 10 PR105D conformations at the TD-B3LYP/6-31G* level. The QM region in the QM/MM calculations consists of residues 230, 231, 232 and closest non-covalent neighbors within 2.5 Å from the central retinal.

Configuration	EE-GMFCC (eV)	QM/MM (eV)	Difference (eV)
1	2.5391	2.4355	0.10
2	2.4953	2.4188	0.08
3	2.4266	2.3329	0.09
4	2.4404	2.4168	0.02
5	2.5474	2.5006	0.05
6	2.6008	2.4888	0.11
7	2.5772	2.5649	0.01
8	2.5572	2.4957	0.06
9	2.4828	2.4572	0.03
10	2.3387	2.4065	-0.07
MUD[a]			0.06

[a]MUD denotes the mean unsigned deviation.

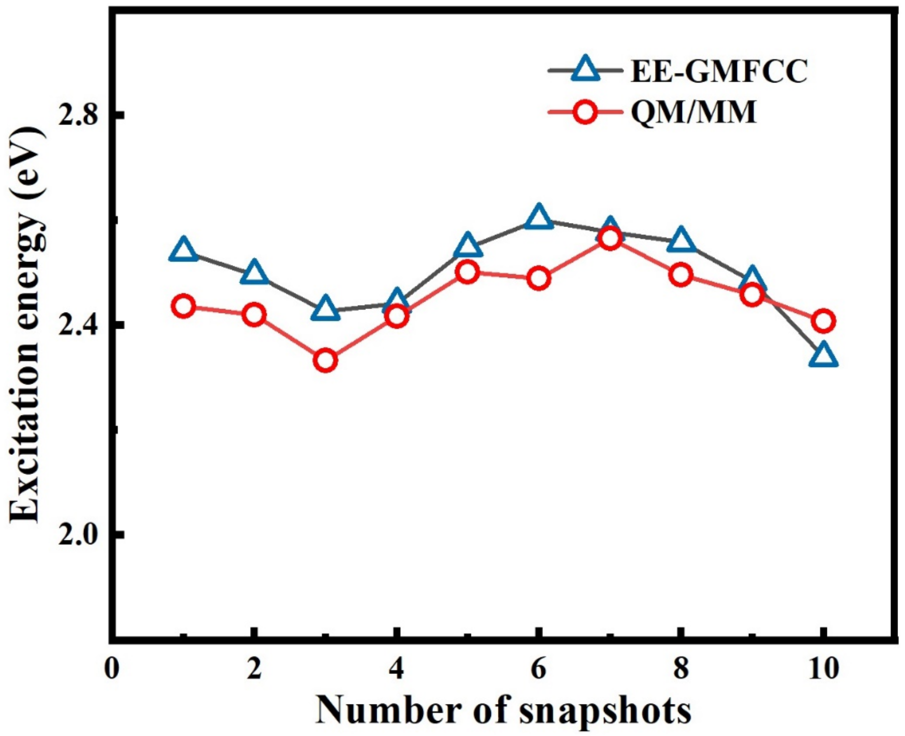

Figure S3. Calculated excitation energy comparison for 10 selected PR105D configurations between the EE-GMFCC approach and the traditional QM/MM method at the TD-B3LYP/6-31G* level.

4. The convergence test of the EE-GMFCC calculations.

Table S2 Average excitation energies of PR105L calculated by the EE-GMFCC method at the TD-B3LYP/6-31G* level.

System	2B (eV)	2B (nm)
PR105L (8th ps)[a]	2.3353	531
PR105L (10th ps)[b]	2.3781	522

[a]Average excitation energy of PR105L calculated using 100 snapshots from the 8th ps QM/MM MD simulation.
[b]Average excitation energy of PR105L calculated using 100 snapshots from the 10th ps QM/MM MD simulation.

5. Excitation energy distributions of 100 conformations of PR105Q and its 9 mutants.

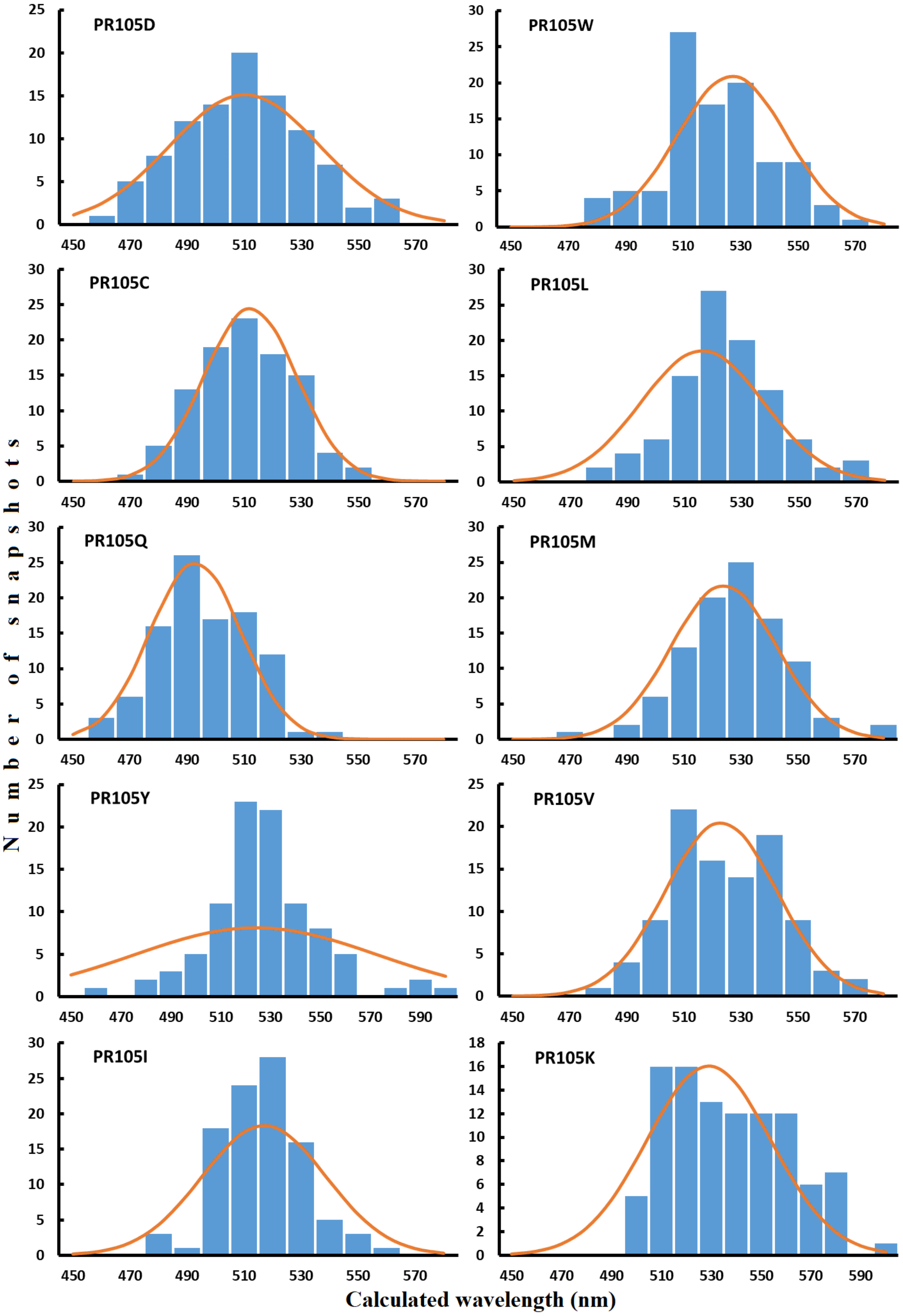

Figure S4. Distribution of calculated excitation wavelengths of PR105Q and its 9 mutations using EE-GMFCC (two-body QM corrections were included) for 100 snapshots from QM/MM MD simulations. The X and Y axes represent the calculated wavelength (nm) and number of snapshots, respectively.

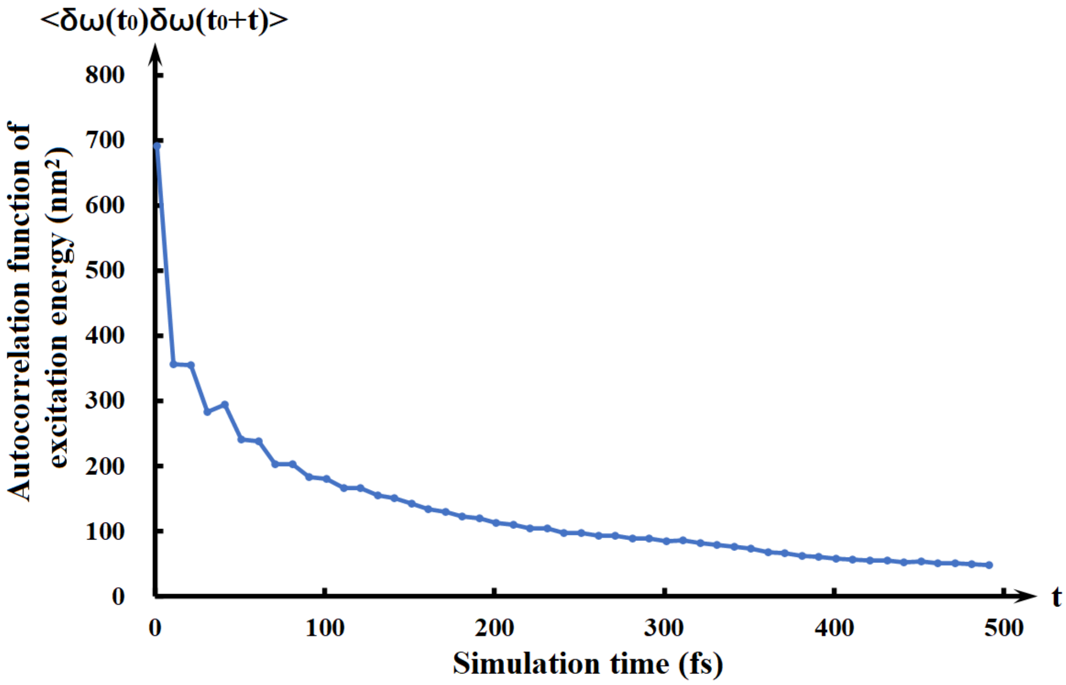

Figure S5. Autocorrelation function of the excitation energies ($\delta\omega(t)= \omega(t)-\overline{\omega(t)}$) for PR105D.

We calculated the time autocorrelation function of the excitation energies for PR105D (see Figure S5). The results show that the decorrelation time (reaching 1/(2e) of the initial value) is about 150 fs. This timescale covers several periods of C=C stretches, which are known to strongly modulate retinal's excitation energies via a bond length alternation coordinate.[2] Therefore, we have 7 independent samples from the 1 ps QM/MM MD simulation trajectory, and the standard error is the standard deviation divided by $\sqrt{7}$ (see Figure S6). The autocorrelation function supports our use of a 1 ps QM/MM MD simulation for obtaining the average excitation energies within our desired accuracy of ±10 nm. In addition, comparison to a different 1 ps of averaging supports our 1-ps sample as being representative (see Table S2). The approach we took is thus adequate to get an average excitation energy that can show the general trend with respect to mutation. It is worth noting that the number of uncorrelated configurations needed to obtain statistically converged averaged absorption energies is expected to be higher for large systems[3-7]. Longer QM/MM MD simulations for proteins are thus needed to sample a sufficiently large number of uncorrelated configurations. Research along this direction will be carried out in future studies.

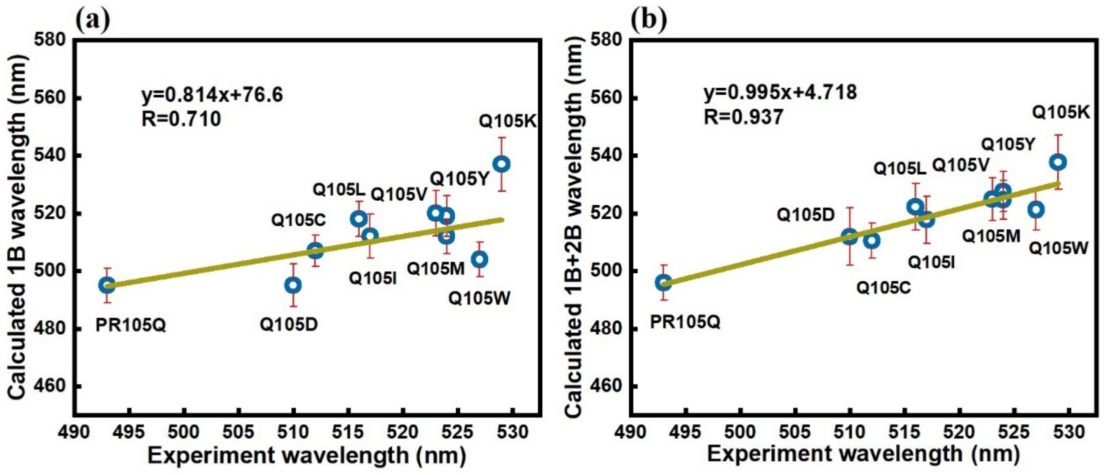

Figure S6. The correlation between the experimental absorption wavelengths of different mutations of PR105Q and calculated results using EE-GMFCC (with standard errors). The 1B and 1B+2B results are both provided. (a) 1B: only the one-body QM interactions are included. (b) 1B+2B: both the one-body and two-body QM interactions are included (see Eq. 4).

6. Correlations between the calculated electric fields (with the AMBER and PPC charge models) and excitation energies calculated by EE-GMFCC, and experimental excitation energies.

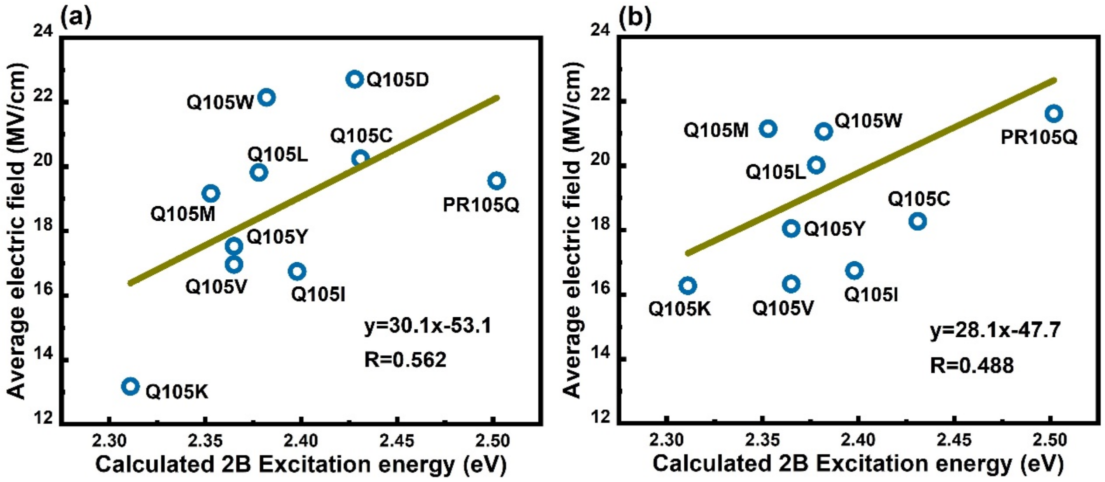

Figure S7. Correlation between the average electric fields of 100 snapshots (using the AMBER ff14SB (a) and PPC (b) charge models, respectively) taken from QM/MM MD simulations of PR105Q and its 9 mutants, and calculated excitation energies using EE-GMFCC (with the two-body quantum mechanical (2B QM) correction).

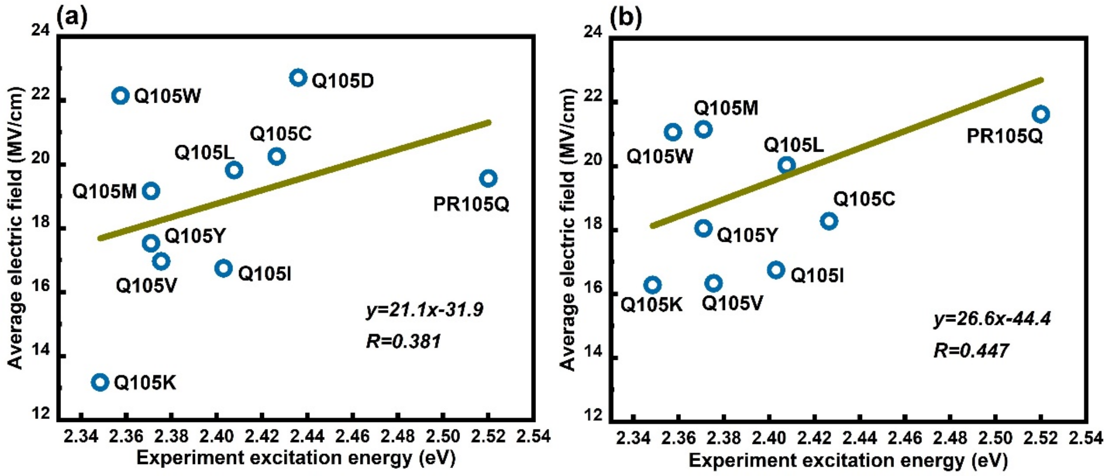

Figure S8. Correlation between the average electric fields of 100 snapshots (using the AMBER ff14SB (a) and PPC (b) charge models, respectively) taken from QM/MM MD simulations of PR105Q and its 9 mutants, and experimental excitation energies.

References:

1. Jin, X.; Glover, W. J.; He, X., Fragment Quantum Mechanical Method for Excited States of Proteins: Development and Application to the Green Fluorescent Protein. *J. Chem. Theory Comput.* **2020,** 16, 5174-5188. doi:10.1021/acs.jctc.9b00980.
2. Yu, J. K.; Liang, R.; Liu, F.; Martinez, T. J., First-Principles Characterization of the Elusive I Fluorescent State and the Structural Evolution of Retinal Protonated Schiff Base in Bacteriorhodopsin. *J. Am. Chem. Soc.* **2019**, 141, 18193-18203. doi:10.1021/jacs.9b08941.
3. van Gunsteren, W. F.; Huenenberger, P. H.; Mark, A. E.; Smith, P. E.; Tironi, I. G., Computer simulation of protein motion. Computer Physics Communications 1995, 91, (1-3), 305-319. doi:10.1016/0010-4655(95)00055-K.
4. Georg, H. C.; Coutinho, K.; Canuto, S., Solvent effects on the UV-visible absorption spectrum of benzophenone in water: a combined Monte Carlo quantum mechanics study including solute polarization. J Chem Phys 2000, 113, (20), 9132–9139. doi:10.1063/1.2426346.
5. Ali, A.; Le, T. T. B.; Striolo, A.; Cole, D. R., Salt Effects on the Structure and Dynamics of Interfacial Water on Calcite Probed by Equilibrium Molecular Dynamics Simulations. The Journal of Physical Chemistry C 2020, 124, (45), 24822-24836. doi:10.1021/acs.jpcc.0c07621.
6. Manzoni, V.; Lyra, M. L.; Gester, R. M.; Coutinho, K.; Canuto, S., Study of the optical and magnetic properties of pyrimidine in water combining PCM and QM/MM methodologies. Phys Chem Chem Phys 2010, 12, (42), 14023-33. doi:10.1039/c0cp00122h.
7. Glover, W. J.; Larsen, R. E.; Schwartz, B. J., Simulating the formation of sodium:electron tight-contact pairs: watching the solvation of atoms in liquids one molecule at a time. J Phys Chem A 2011, 115, (23), 5887-94. doi:10.1021/jp1101434.
